# Supplementary material for: Evaluation of ultraviolet irradiation at 254 nm and 222 nm in inactivating human noroviruses on surfaces
Source: Appl Environ Microbiol. 2026 Apr 20;92(5):e02514-25. doi: 10.1128/aem.02514-25 (PMC13188894; doi:10.1128/aem.02514-25)
Supplement: Supplemental material — Supplemental methods and results. [file aem.02514-25-s0001.docx]

**Supplementary information**

**S1. Details in materials and methods**

**Zebrafish husbandry and embryo maintenance.** Wild-type adult zebrafish (*Danio rerio*) were housed in the Comparative Medicine facility at National University of Singapore, with water temperature maintained at 28 ± 2°C and a 14:10 h light/dark cycle. Fertilized embryos were obtained from adults placed in mating cages and subsequently maintained in E3 buffer (pH = 7.2; 5 mM NaCl, 0.17 mM KCl, 0.33 mM CaCl_2_, 0.33 mM MgSO_4_) at 29.5 ± 0.5°C. All experimental procedures involving zebrafish were approved by the Institutional Animal Care and Use Committee (IACUC) of National University of Singapore.

**Microinjection of virus into zebrafish embryos.** Referring to H. Tan Malcolm Turk et al. (1), zebrafish embryos were firstly transferred to a petri dish containing a 1.5% agarose layer imprinted with a mold forming six rows of V-shaped grooves. Under a stereo microscope (Zeiss Stemi 508, Carl Zeiss Microscopy GmbH, Jena, Germany), 3 nL of hNoV sample was microinjected into the yolk of each embryo using a pulled borosilicate glass capillary needle (Drummond Scientific Co., Broomall, PA, USA). Post-injection, the embryos were transferred to a Petri dish containing E3 buffer and maintained at 29.5 ± 0.5°C. Embryos or larvae were monitored daily post-infection (dpi) to assess developmental progression. Non-viable individuals were removed, and the medium was refreshed daily.

**Standard curve construction for RT-qPCR methods.** For hNoV GII detection, double-stranded DNA fragments containing the target primers-probe binding sites were synthesized and cloned into the pGEM-T Vector (Promega) to generate the hNoV GII plasmid. The purification of plasmids was conducted using the Plasmid Midi Kit (Qiagen) and quantified spectrophotometrically at 260 nm using the BioDropDuo™ spectrophotometer (BioDrop, Cambridge, UK). Ten-fold serial dilutions of plasmid were used to construct standard curves and titer hNoV GII viruses. For TV and MS2, standard curves were constructed in each trial using 10× serial dilution of controls.

**S2. Recovery rates of virus from different surfaces**

Table S1 The recovery rates of hNoVs or surrogates on different surfaces in hydrated and dried inocula

| Surfaces | Inoculum | Virus type | Recovery rate (%) |
| --- | --- | --- | --- |
| Petri dish | Hydrated | All | 96.8 – 99.3 |
| Stainless-steel plate | Hydrated | hNoV GII.17 | 98.9 ± 0.5 |
|  |  | MS2 | 97.4 ± 0.9 |
|  | Dried | hNoV GII.17 | 50.1 ± 3.6 |
|  |  | MS2 | 46.5 ± 2.2 |
| Porcine ear skin | Hydrated | hNoV GII.17 | 98.7 ± 0.3 |
|  |  | MS2 | 98.2 ± 1.2 |
|  | Dried | hNoV GII.17 | 8.6 ± 0.9 |
|  |  | MS2 | 6.3 ± 1.7 |

**S3. Details in measurement of incident fluence rate and emission spectra of UV lamps**

**The measurement of incident fluence rate of UV irradiations.** The potassium iodide-iodate (KI/KIO_3_) actinometry is widely used in measurement of UVC radiation due to the theoretical total absorbance below 290 nm in theory (2). The actinometry is based on the following reaction: 8I^-^ + IO_3_^-^ + 3H_2_O + *hv* → 3I_3_^-^ + 6OH^-^. The generation of triiodide (I_3_^-^) can be quantified spectrophotometrically by its characteristic absorbance peak at 352 nm (3), to proportionally reflect the incident UV fluence rate.

The procedures were adapted from R. Yin et al. (4) with minor modifications. A solution containing 0.577 M of KI and 0.1 M of KIO_3_ was prepared, transferred to a Petri dish, and exposed to UV 222 or UV 254 irradiations for predetermined time intervals. Subsequently, the absorbance at 352 nm was measured using a UV-Vis spectrophotometer (UV mini-1240, Shimadzu Corporation, Kyoto, Japan). Control samples were measured prior to irradiation to account for background absorbance. The equation of incident fluence rate (*E_0_*) is as follows:

$E_{0}=\frac{\frac{\text{ΔOD}_{352}}{{t\mathcal{\times E}}_{I_{3}^{-}}}\times V\times U_{\lambda}}{\Phi\times S}$ Eq. S1

$U = \frac{hcN_{A}}{}$ Eq. S2

where *ΔOD_352_* is the difference of the absorbance of KI/KIO_3_ solution at 352 nm after UV irradiation (cm^-1^); *t* is the irradiation time (s); $\mathcal{E}_{I_{3}^{-}}$is 26400 (M • cm)^-1^, the molar absorption coefficient of *I_3_^-^*; *V* is 15 mL, the solution volume for each treatment; *Φ* is the quantum yields of at the respective UV wavelengths (*Φ222* = 0.92, *Φ254* = 0.74); *S* is the irradiation area of the Petri dish, 69.36 cm^2^; *U_λ_* is the molar photon energy of the UV radiation at the particular wavelength (J einstein^-1^); *h* is the Planck constant (6.63 × 10^–34^ J • s); *c* is the speed of light (3.00 × 10^8^ m • s^–1^); *N_A_* is the Avogadro number (6.02 × 10^23^ mol^–1^); and *λ* is the wavelength in meters (2.22 × 10^11^ or 2.54 × 10^11^ m). According to the complete fluence response curve after measurement, *E_0_* could be calculated, which is the coefficient in function of fluence and time (5).

**The correction of the average fluence rate.**

1. Water factor (WF) corrects for the attenuation of the incident fluence rate due to the water absorption in the irradiated column according to Beer-Lambert law (4, 6):

$E_{0,ave}=E_{0}\cdot WF$ Eq. S3

$WF=\frac{1-{10}^{-al}}{al\cdot ln(10)}$ Eq. S4

$l=\frac{V}{\pi r^{2}}$ Eq. S5

where *a* is the corresponding decadic absorption coefficient (cm^–1^) at 222 nm or 254 nm; *l* is the vertical path length (cm) of the water in the petri dish; *V* is the solution volume (mL); *r* is the radius of the Petri dish (4.5 cm).

2. Reflection factor (*RF*) and petri factor (*PF*) have been already taken into considerations by actinometer, thus there is no need to offer extra corrections for *PF* and *RF* by using this KI/KIO3 actinometry (7). Other correction parameters, including divergence factor, as well as the influence of water depth, can be ignored in this study, which is similar to the experimental set-up of Q. Bai et al. (7).

**The emission spectra of UV 222 and UV 254 lamps.** Within UV region (200 to 400), far-UVC 222 nm lamp with filter and UV 254 lamp were characterized by an ST Spectrometer (ST01596, Ocean Optics, USA) as shown in Fig. S1.

Fig. S1 The spectral emission within UV range for the UV lamps with the wavelength of 222 and 254 nm.

**S4. Synergistic effect of UV 222 and 254.**

**Materials and methods.** Firstly, 10 μL droplets of virus samples were deposited in petri dishes and exposed to UV irradiation at doses of 7 and 70 mJ/cm^2^. Treatments were applied using either UV 222 or UV 254 individually, or in a sequential combination (UV 222+254), where droplets were first exposed to UV 222 (7 mJ/cm^2^), followed immediately by UV 254 (7 mJ/cm^2^). The combined treatments were used to investigate synergistic effect of the two wavelengths. After UV treatments, the droplets were recollected by pipetting into 1.5-mL tubes for RNA extraction or infectivity assays.

**Results and discussion.** A synergistic effect occurs when the combined action of two treatments yields a result that is greater than the sum of their individual effects. In this study, the synergy effect of combined treatment was evaluated through synergy coefficient (*S*), the ratio between log reduction of combined UV treatments (UV 222+254) and the sum of log reductions of UV 222 and UV 254 (8). If *S* > 1 and *p* < 0.05, it indicates synergistic effect exists. Otherwise, there is no synergy of the two treatments.

As shown in Fig. S2, the effects of sequentially combined treatments of the two wavelengths did not significantly exceed additive effects, as evidenced by the infectivity loss and PCR results of hNoV strains and surrogates (*p* > 0.05). Due to the detection limit of scoring approach, it was impossible to compare the infectivity losses of combined and additive effects for hNoV strains. From infectivity results (Fig. S2A) of TV and MS2, *S* in both groups were less than 1, while the difference within each strain was proved to be insignificant (*p* > 0.05). Notably, as shown in Fig. S2B, only GII.2[P16] from RT-qPCR results demonstrated synergistic effect (*S* > 1 and *p* < 0.05). However, TV showed the opposite trend, with *S* < 1 and *p* < 0.05 (Fig. S2B). Collectively, no synergistic effect was observed when UV 222 and 254 nm irradiations were combined in use for the inactivation of hNoVs.


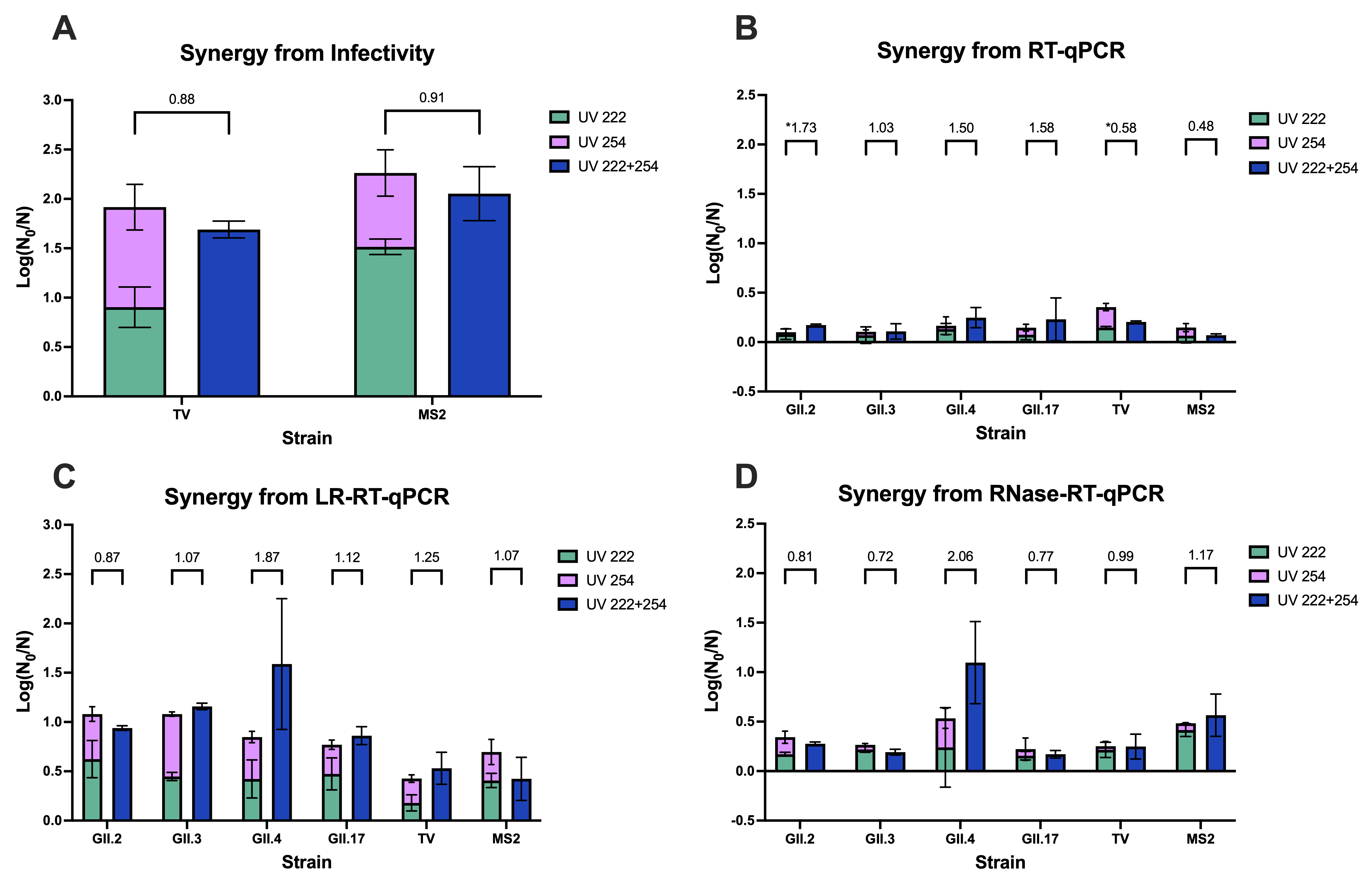


Fig. S2 Synergistic effects of sequentially combined treatments (UV 222+254) in comparison with additive effects of UV 222 and UV 254 against hNoVs (GII.2[P16], GII.3[P12], GII.4 Sydney[P16] and GII.17[P31]) and surrogates (TV and MS2) through infectivity assays (A; TCID_50_ for TV, PFU for MS2), RT-qPCR (B; in RT-qPCR units), LR-RT-qPCR (C; in LR-RT-qPCR units) and RNase-RT-qPCR (D; in RT-qPCR units). *: *p* < 0.05. The number over each pair comparison means the synergy coefficient (*S*), the ratio between log reduction of UV 222+254 and sum of log reduction of individual treatments of UV 222 or UV 254 (8). If *S* > 1 and *p* < 0.05, the synergy exists. Otherwise, no synergy.

Note: Specially for TV, as the effect of UV 222 was incomparable to that of 254 nm, the dose of UV 222 was increased to 70 mJ/cm^2^ for both individual (70 and 7 mJ/cm^2^) and combined treatments (70+7 mJ/cm^2^). Other treatments were administered at the doses of 7 mJ/cm^2^ for individual or 70+7 mJ/cm^2^ for combined treatments.

In the context of microbial inactivation, a strategic combination of two or more disinfection or treatment methods is commonly explored to enhance effectiveness and reduce treatment intensity. Such an approach aims to conserve energy costs, mitigate equipment degradation, and ensure safety (9). Synergistic effect of dual-wavelength UVC irradiation of 222 and 254 nm, which has been observed in bacterial inactivation (10), were not achieved in the treatment of hNoVs. This was possibly due to the overlapping action of UV 222 and UV 254 on the viral RNA, despite of the additional capsid damage induced by UV 222.

**S5. Mutation results from hNoV passaging until P24**

In control passaging line #1, GII.4 Sydney[P16] was continuously passaged to P12, P18 and P24 to evaluate the cumulative effects of host impact over extended replication cycles (Fig. S3).

When control P6 #1 was further passaged to P24 (Fig. S3), the number of non-synonymous SNVs progressively increased from two in P6, to seven in P12 and P18, reaching ten in P24. Importantly, three SNVs at positions 5145, 5406 and 5661 nt, accumulated within ORF2 region, which encodes VP1, the major virus capsid protein and contains important determinants of antigenicity (11). An additional SNV at 4407 nt was identified within RdRp coding region, where mutations may contribute to the emergence of variants bearing a novel P-type (12). In addition to the rising numbers of non-synonymous SNVs, variant frequencies also increased over passaging. For instance, SNVs at positions 1450 and 5145 nt, initially presenting below 30% frequency in P12, surpassed 50% in P18. The non-synonymous SNVs identified in P6 (at 3627 and 4471 nt) persisted throughout all subsequent generations. Ultimately, four new SNVs as non-synonymous substitutions emerged in P24 at positions 498, 5661, 7105 and 7327 nt.

**
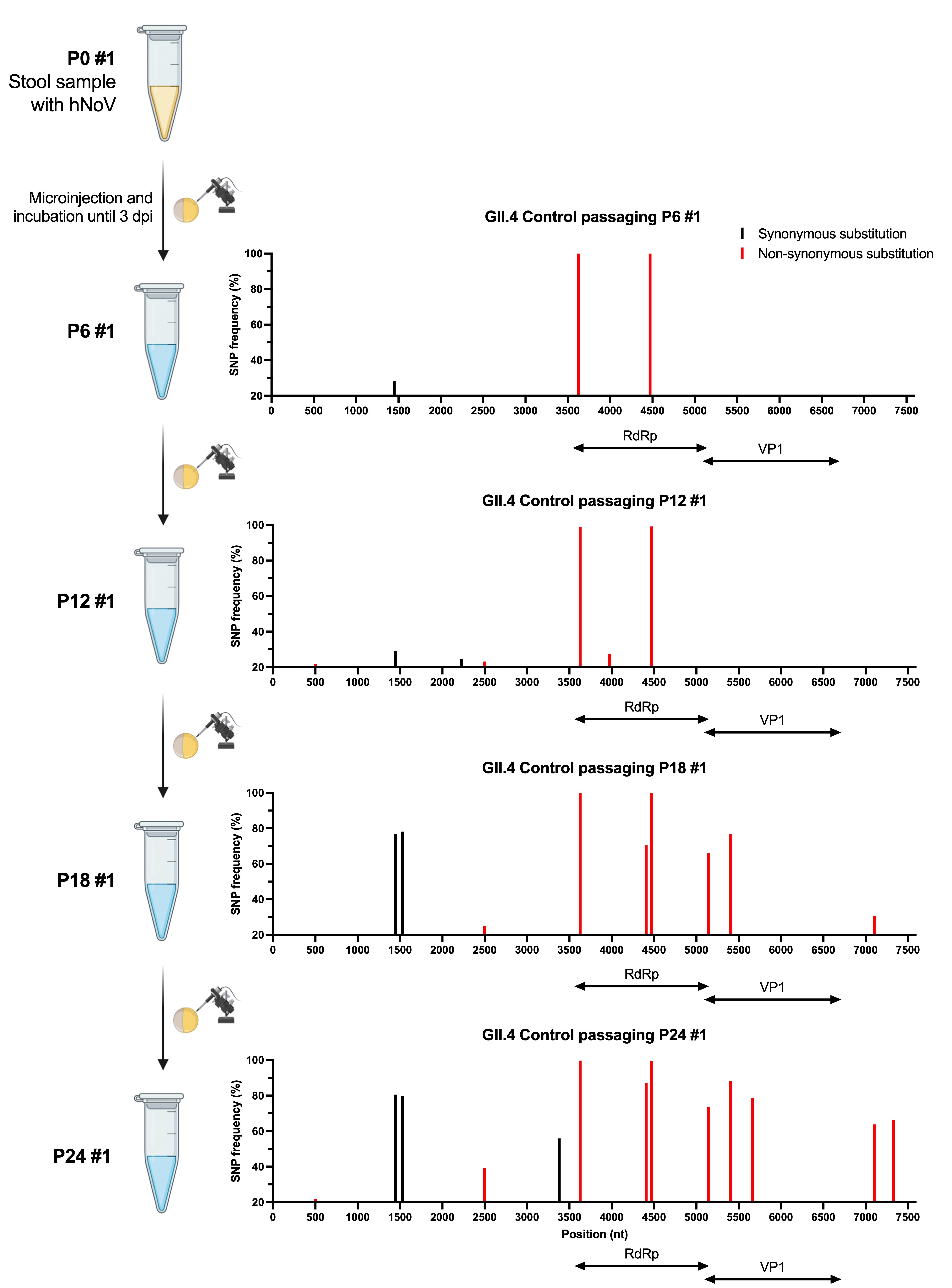
**

Fig. S3 Illustration of control passaging line #1 of hNoV GII.4 Sydney[P16] to the 24th generation (P24), together with SNPs identified in 6th (P6), 12th (P12), 18th (P18) and 24th (P24) generations. Two variant determinant regions: RNA-dependent RNA polymerase (RdRp) is located within 3558–5087 nt and open reading frame 2 in 5071–6693 nt. Red columns are SNVs with non-synonymous substitution; black columns are SNVs with synonymous substitution; The threshold of SNP frequency is 20%. Absolute virus titers by RT-qPCR: P0 #1 (6.4 log genome copies/μL); P6 #1 (8.9 log genome copies/μL); P12 #1 (8.8 log genome copies/μL); P18 #1 (8.4 log genome copies/μL); P24 (8.6 log genome copies/μL).

References:

1. Tan Malcolm Turk H, Gong Z, Li D. 2023. Use of Zebrafish Embryos To Reproduce Human Norovirus and To Evaluate Human Norovirus Infectivity Decay after UV Treatment. Applied and Environmental Microbiology 89:e00115-23.

2. Rahn RO. 1997. Potassium Iodide as a Chemical Actinometer for 254 nm Radiation: Use of Iodate as an Electron Scavenger. Photochemistry and Photobiology 66:885-885.

3. Bolton JR, Stefan MI, Shaw P-S, Lykke KR. 2011. Determination of the quantum yields of the potassium ferrioxalate and potassium iodide–iodate actinometers and a method for the calibration of radiometer detectors. Journal of Photochemistry and Photobiology A: Chemistry 222:166-169.

4. Yin R, Zhang Y, Wang Y, Zhao J, Shang C. 2024. Far-UVC Photolysis of Peroxydisulfate for Micropollutant Degradation in Water. Environmental Science & Technology 58:6030-6038.

5. Bolton James R, Linden Karl G. 2003. Standardization of Methods for Fluence (UV Dose) Determination in Bench-Scale UV Experiments. Journal of Environmental Engineering 129:209-215.

6. Sperle P, Mirlach A, Linden K, Hübner U, Drewes JE. 2023. An actinometric method to characterize performance of reflecting UVC reactors used for water treatment. Water Research 230:119543.

7. Bai Q, Wu Q-Y, Ye B, Wu Y-P, Lee J-W, Lee M-Y, Wang W-L. 2024. Assessing excimer far-UVC (222 nm) irradiation for advanced oxidation processes: Oxidants photochemistry and micropollutants degradation. Water Research 267:122505.

8. Sun Z, Li M, Wu Z, Wang Y, Blatchley ER, III, Xie T, Qiang Z. 2025. Water Disinfection with Dual-Wavelength (222 + 275 nm) Ultraviolet Radiations: Microbial Inactivation and Reactivation. Environmental Science & Technology 59:1448-1456.

9. Le TV, Dang T-LT, Imai T. 2023. Synergistic bactericidal activity of ultraviolet radiation, ozone, and liquid-thin-film technology against Escherichia coli in water. Water Supply 23:884-894.

10. Kang J-W, Kang D-H. 2018. The Synergistic Bactericidal Mechanism of Simultaneous Treatment with a 222-Nanometer Krypton-Chlorine Excilamp and a 254-Nanometer Low-Pressure Mercury Lamp. Applied and Environmental Microbiology 85:e01952-18.

11. de Graaf M, van Beek J, Koopmans MPG. 2016. Human norovirus transmission and evolution in a changing world. Nature Reviews Microbiology 14:421-433.

12. Chhabra P, de Graaf M, Parra GI, Chan MC-W, Green K, Martella V, Wang Q, White PA, Katayama K, Vennema H, Koopmans MPG, Vinjé J. 2019. Updated classification of norovirus genogroups and genotypes. Journal of General Virology 100:1393-1406.
